# Can lymphocytes serve as a predictor of response to preoperative chemoradiation therapy for locally advanced rectal cancer?

**DOI:** 10.3389/fonc.2023.1138299

**Published:** 2023-04-03

**Authors:** Myroslav Lutsyk, Tarek Taha, Salem Billan

**Affiliations:** ^1^ Ha’Emek Medical Center, Afula, Israel; ^2^ The Baruch Padeh Medical Center, Poriya, Poriah, Israel; ^3^ Rambam Health Care Campus, Haifa, Israel

**Keywords:** lymphopenia, neoadjuvant chemo(radio)therapy, pathological complete response (pCR), tumor response, rectal adenocarcinoma

## Abstract

**Introduction:**

The aim of this study is to identify factors that may predict the response of locally advanced rectal cancer tumors (LARC) to neoadjuvant chemoradiotherapy (CRT) and to evaluate the effect of circulating lymphocytes on pathological tumor response.

**Methods:**

This retrospective study included neoadjuvant CRT-treated, LARC-diagnosed patients at the Rambam Health Care Campus in Haifa, Israel. CHAID analysis, t-test, χ^2^ test, and ROC curve analyses were performed to explore the association between pathological complete response (pCR) and several factors including patient demographics, tumor characteristics, type of treatment, and levels of circulating lymphocytes measured on a weekly basis.

**Results:**

Out of 198 patients enrolled in the study, pCR was achieved in 50 patients (25%). ROC curve and CHAID analyses showed that absolute lymphopenia was significantly associated with lower pCR rates (*p*=0.046 and p=0.001, respectively). Other factors that were found to have a significant impact were radiation therapy type (*p*=0.033) and tumor distance from the anal verge (*p*= 0.041).

**Conclusion:**

An absolute decrease in the level of circulating lymphocytes during preoperative CRT to LARC is associated with poorer tumor response to treatment and thus may serve as a predictive biomarker for treatment resistance.

## Introduction

Rectal cancer is the seventh most common cancer in the world accounting for 730,000 new cases per year (1). The incidence is higher in men and more common in adults with the average age at diagnosis being 63 years (2). The disease is associated with the Western lifestyle and its incidence is greater in developed countries. Mortality rates, however, are higher in developing countries, which may reflect the limited health infrastructures in those nations and demonstrate the impact of treatment on survival and life expectancy. By 2030, the global burden of colorectal cancer is expected to rise by 60%, when estimates suggest there will be 2.2 million new cases and 1.1 million deaths (3).

Treatment for rectal adenocarcinoma is determined by the clinical stage of the disease and the location of the tumor in the rectum. In its earliest stages, the standard treatment is local excision, which can be sufficient, without removal of lymph nodes or any further action. The objective is tumor removal with free resection margins that minimizes the chances of local recurrence, which are considered relatively high due to the cancer’s anatomical location in the pelvis and its proximity to other organs and structures (4).

According to guidelines issued by the National Comprehensive Cancer Network (NCCN), the American Society of Clinical Oncology (ASCO), and the European Society for Medical Oncology (ESMO) (5–7), the standard of care for locally advanced disease (stages II-III (cT3-4N0 or cTxN1-2)) is the provision of preoperative CRT and radical resection of the rectum – total mesorectal excision (TME). The main surgical approaches to the treatment of tumors at these stages are low anterior resection (LAR), which includes the preservation of sphincters, or abdominoperineal resection (APR) in lower positioned tumors, located within 6 cm from the anal verge, which do not allow preservation of the anus and sphincters (8).

Prior to surgery, neoadjuvant radiation therapy can be given as a short course (total dose of 25Gy using 5Gy fractions for 5 days) or as a long course (total dose of 50-54Gy using 1.8-2.0Gy fractions for 5 weeks), in conjunction with 5-fluorouracil-based chemotherapy or capecitabine, as studies have shown that the addition of chemotherapy significantly improves local control rates (9, 10). The surgery may be performed immediately or 6-8 weeks after neoadjuvant treatment ends.

The goal of neoadjuvant therapy is to reduce the size of the tumor (downgrading) and/or to reduce the stage of the disease (downstaging) to allow more efficient resection with free margins, thus lowering the chances of local recurrence (11, 12). Treatment can lead to downstaging in 50-60% of patients and even pathological complete response (pCR) in 10-20% of cases (13). pCR is defined as the absence of tumor cells in the rectum or lymph nodes and their replacement by fibrotic tissue as observed microscopically in the surgical sample obtained during the surgery (ypT0N0M0). Therefore, pCR is an important prognostic factor in the assessment of rates of recurrence, overall survival (OS), and disease-free survival (DFS) (14, 15).

A wide range of factors are known to affect tumor response to CRT, such as pathological and clinical tumor stage, distance from the anal verge, and the time between the end of neoadjuvant therapy and surgery (12). Studies have also shown that blood lymphocyte levels before, during, and after neoadjuvant therapy predict prognosis and are positively associated with pCR (16, 17). Accordingly, it is greatly important to evaluate measurable variables that may predict treatment efficacy and the likelihood of a pCR. Patients predicted to have good treatment response may cause oncologists to refine or alter their decisions (e.g., opting to intensify preoperative chemotherapy treatment, provide less radiation therapy, etc.), which might result in large differences in the adverse events profile.

Twenty-five percent of the bone marrow of elderly adults’ reserve is contained in the pelvic bones, which are considered a metabolically active focus. It is also known that of all blood cells, lymphocytes are the most radiation-sensitive, having an LD50 of 2Gy. Secondary lymphopenia caused by radiation therapy is a common phenomenon among oncology patients in general and patients with rectal cancer in particular (18). The decrease in lymphocyte levels is exponential and begins after the first week of treatment (19, 20). Although it is usually an acute side effect that resolves about 3 months after treatment end, several studies have shown that lymphopenia is a poor prognostic factor for progression-free survival (PFS) and OS in various tumors (16, 21). It is hypothesized that lymphocytes play a significant role in the anticancer activity of the immune system, as a greater density of T cells in the tumor bed has been shown to be associated with higher OS and DFS (22).

The hypothesis of the present investigation is that a decrease in the level of lymphocytes during preoperative neoadjuvant CRT treatment of rectal cancer predicts lower responsiveness of the tumor to treatment.

## Materials and methods

This retrospective study was approved by the Ethics Committee of the Rambam Health Care Campus in Haifa, Israel (0315-19-RMB). The inclusion criteria included patients referred to Rambam’s Radiation Therapy Unit between September 2015 to January 2020 following diagnosis of rectal adenocarcinoma by histopathological examination, clinical stage IIA-IIIC, per the TNM v8. T-stage was determined using transrectal ultrasonography and pelvic MRI, and N-stage was assessed using MRI and PET-CT. Patients who were treated with induction or consolidation chemotherapy before or after a chemoradiation course, which signifies a total neoadjuvant treatment (TNT) approach, were excluded from this study.

### Radiation therapy characteristics

Each patient was administered a total radiation therapy dose of 50Gy to the tumor volume in daily 2Gy increments *via* simultaneous integrated boost (SIB), along with 45Gy in daily 1.8Gy increments to pelvic lymph nodes. Each treatment was planned using the Monaco Treatment Planning System (TPS) and delivered 5 times a week for 5 weeks. The volumetric modulated arch therapy (VMAT) technique was used to deliver 6- or 10- MV photon beam energies with Agility HD MLC transmission optimization. Gross tumor volume (GTV), visualized on a CT-based simulation with fusion of pretreatment MRI or PET-CT images on TPS, was contoured by a radiation oncology expert, revised by radiology and nuclear medicine expert, and approved in a weekly radiation oncology staff meeting. Clinical target volume (CTV) to tumor SIB was contoured by adding 1.5 -2.0 cm around the GTV and adding 0.5 cm around the CTV -planning target volume for the SIB. Pelvic lymph node volume (CTV45) was created by the contouring of mesorectal fat, the presacral lymph nodes 0.5-0.7 cm anteriorly from the ventral aspect of the sacrum, an 1.0-1.5 cm expansion around the internal iliac, and obturator blood vessels. In cases where there was involvement of the anal canal or the explicit pathologic appearance of lymph nodes, the external iliac nodes and/or common iliac lymph nodes were included in the CTV45. An additional expansion of 0.5 cm around CTV45 thereby established the pelvic planning target volume (PTV45). Volume values were measured automatically by radiation therapy TPS software.

### Chemotherapy regimen

Chemotherapy was applied using 5-fluorouracil in a dose of 300 mg/m^2^ for 96 hours weekly or capecitabine at 825 mg/m^2^, given twice a day, 5 days a week during 5 weeks of the radiation treatment.

### Surgery and pathology

Surgery was performed 6-8 weeks after completion of chemoradiation therapy using the total mesorectal excision technique. Resected tissue was examined by a senior pathologist to evaluate the response of the primary tumor and lymph nodes. Complete pathological response was defined as no viable tumor cells in primary tumor tissue and in all resected lymph nodes. Based on the response, two groups of patients were identified: one presenting a pathological complete response (pCR) in both primary tumor and lymph nodes and the other with a less than complete response (no-pCR).

### Blood test

Blood tests were performed weekly on each chemoradiation course, and an absolute lymphocyte count (ALC) was registered. Patients having lymphopenia (<1x10^9^/L) at the time of chemoradiation start were excluded from the study. Further analysis was performed to compare host, tumor, and treatment characteristics between lymphopenic and non-lymphopenic patients.

### Statistical analysis

For the calculation of descriptive and frequency statistics, analyses were performed using IBM SPSS Statistics v.27 software. Crosstab with chi-square tests were used to execute comparisons between the two groups (i.e., with and without pCR). A chi-square test with an independent t-test was carried out to estimate homogeneity between the pCR and non-pCR groups. Presuming nonparametric distribution of observed pCR and lymphopenia, the nonparametric chi-square test was used. To assess the role of chemoradiotherapy-induced lymphopenia in the achievement of pCR, a univariate analysis was used along with age, gender, ethnicity, body weight and height, smoking status, level of tumor in the rectal wall, delivered RT dose, GTV, and PTV45 variables. To evaluate correlations between clinical, blood test, and radiotherapeutic features and to exclude possible collinearity of exploring factors, a factor analysis was performed. The variables included in this analysis were age, GTV, PTV45, level of lower tumor margin, and absolute lymphocyte count at the conclusion of radiotherapy course.

## Results

Between 2015 and 2020, 354 patients were referred to our Radiation therapy Unit for neoadjuvant radiotherapy. After collecting data and excluding patients with an absolute lymphocyte count in their blood samples, 202 patients were enrolled in the study. Four additional patients were excluded due to the presence of synchronous metastasis. Patient demographics and characteristics are presented in **Table 1**. The mean age of the patients at the time of diagnosis was 60.6 ± 11.73 years, with 74 females (37.4%) and 124 males (62.6%); 76 Arab (38.4%) and 122 Jewish (61.6%) patients. There were 150 patients (76%) who were past smokers or had never smoked.

**Table 1:** Demographic, anthropometric, and clinical characteristics of study participants (n=198).

Characteristic	Mean ± SD
Age (years)	60.6 ± 11.73
Height (cm)	167.45 ± 12.3
Weight (kg)	77.65 ± 15.4
Gender (n, %)	
Female	74 (37.4%)
Male	124 (62.6%)
Ethnicity (n, %)	
Arabs	76 (38.4%)
Jews	122 (61.6%)
Smoking (n, %)	
Light smoker	6 (3%)
Heavy smoker	42 (21.2%)
Past smoker	26 (13.1%)
Never smoked	124 (62.6%)
Clinical Stage (n, %)	
IIa	52 (26.3%)
IIb	4 (2%)
IIc	1 (0.5%)
IIIa	4 (2%)
IIIb	115 (58.1%)
IIIc	22 (11.1%)
GTV (cm^3^)	47.81 ± 45
PTV (cm^3^)	1066.14 ± 296.61
Distance from anal verge (cm)	6.79 ± 2.77
Delivered dose (Gy)	49.9 ± 0.74
ALC for last 2 weeks of CRT (10^9^/L)	0.8 ± 0.32
OS (months)	23.56 ± 14.1

SD, standard deviation; GTV, gross tumor volume; PTV, planning target volume; ALC, absolute lymphocyte count; CRT, chemoradiotherapy; OS, overall survival.

Disease in Stage II or Stage III was diagnosed in 57 and 141 patients, respectively. Mean gross tumor volume (GTV) was 47.81 ± 4.5 cm^3^ and PTV was 1066.14 ± 296.61 cm^3^. The Mean absolute lymphocyte count for the two last weeks of chemoradiation was 0.8 ± 0.32 10^9^/L. The follow-up time was in the range of 9 to 78 months, with a mean of 36.5 ± 1.4 months. The mean delivered radiotherapy dose was 49.9 ± 0.74Gy.

**Table 2** presents the pathological outcomes of neoadjuvant chemoradiation therapy.

**Table 2:** Pathological outcome of neoadjuvant chemoradiation (n=198).

Characteristic	N (%)
Stage	
0	58 (29.3%)
I	44 (22.2%)
IIa	42 (21.2%)
III	1 (0.5%)
IIIa	19 (9.6%)
IIIb	21 (10.6%)
IIIc	13 (6.6%)
Achieved pCR	
No pCR	145 (73.2%)
pCR	53 (26.8%)
Recurrence	
No recurrence	172 (86.9%)
Recurrent disease	26 (13.1%)
Viability	
Alive	183 (92.4%)
Deceased	15 (7.6%)

pCR; pathological complete response.

Taking into consideration that the current TNM system permits a TisN0M0 case to be classified as a Stage 0 disease - 58 patients (28.7%) were diagnosed as pathological Stage 0. Six cases (6.2%) presented a near- complete response (maximal treatment response, MTR) to delivered treatment, where only several islets of viable tumor cells were found on pathological examination. Those MTR cases were formally rendered as non-pCR patients, resulting in pCR in 53 cases (26.2%) (**Table 2**). During the observation period, there were 29 patients (14.4%) who experienced local or distant recurrences (detected by imaging and/or endoscopic procedures during follow up), while 17 patients (8.4%) died. The results after splitting the patient groups according to observed lymphopenia are presented in **Tables 3** and **4**. The chi- square test showed a significant difference between observed and expected rates of lymphopenia and pCR (p<0.001). Further crosstabulation of T- and N-downstaging rates observed in patients with and without lymphopenia showed statistical significance in the lymph node response rate (p=0.029). The primary tumor response rate was not significant between the two groups.

**Table 3:** Descriptive statistics of lymphopenic vs. non-lymphopenic participants (n=198).

Characteristic	No lymphopenia (n=50)	Lymphopenia (n=148)	T-test (*p*)
Age (years)	60.8 ± 1.5	60.5 ± 0.09	
Height (cm)	167.08	167.57	0.039
Weight (kg)	78.82	77.26	
Gender n (%)			
Female	18 (36%)	56 (37.8%)	
Male	32 (64%)	92 (62.2%)	
Ethnicity n (%)			
Arabs	23 (46%)	53 (35.8%)	
Jews	27 (54%)	95 (64.2%)	
Smoking n (%)			
Light smoker	2 (4%)	4 (2.7%)	
Heavy smoker	11 (22%)	31 (20.9%)	
Past smoker	8 (16%)	18 (12.2%)	
Never smoked	29 (58%)	95 (64.2%)	
GTV (cm^3^)	38.65	50.9	
PTV (cm^3^)	973.94 ± 2	1097.28 ± 2	0.05
Distance from anal verge (cm)	7.2	6.65	
Delivered dose (Gy)	49.908	50.003	
ALC for last two weeks of CRT (10^9^/L)	1.24 ± 0.03	0.65 ± 0.01	<0.000
DFS (months)	31.4 ± 1.9	28.5 ± 1.7	0.02

SD, standard deviation; GTV, gross tumor volume; PTV, planning target volume; ALC, absolute lymphocyte count; CRT, chemoradiotherapy; DFS, disease-free survival.

Results are shown as mean ± SD or n (%0) as specified.

**Table 4:** Clinical characteristics of non-lymphopenic vs. lymphopenic participants (n=198).

Characteristic	No lymphopenia (n=50)	Lymphopenia (n=148)
Clinical T stage		
T2	1 (2%)	3 (2%)
T3	48 (96%)	133 (89.9%)
T4b	1 (2%)	12 (8.1%)
Clinical N stage		
N 0	17 (34%)	40 (27%)
N 1	27 (54%)	92 (62.2%)
N 2	6 (2%)	16 (10.8%)
Cinical stage		
IIa	16 (32%)	36 (24.3%)
IIc	1 (2%)	4 (2.7%)
IIIa	1 (2%)	3 (2%)
IIIb	26 (52%)	89 (60%)
IIIc	6 (12%)	16 (10.8%)
Achieved pCR		
pCR	20 (40%)	33 (22.3%)
no pCR	30 (60%)	115 (77.7%)
Recurrence		
No recurrence	47 (94%)	125 (84.5%)
Recurrent disease	3 (6%)	23 (15.5%)
Viability		
Alive	47 (97%)	138 (91.9%)
Deceased	5 (3%)	12 (8.1%)

pCR, pathological complete response.

Results are shown as n (%).

Lymphopenia was observed in 148 patients (75%) having a mean ALC of 0.65 ± 0.01, 10^9^/L while in 50 (25%) the mean ALC was 1.24 ± 0.03, 10^9^/L (t-test, p<.0001) (**Table 3**). Between non-lymphopenic and lymphopenic patients, the t-test showed significant differences in baseline ALC levels, measured a week before treatment start (p<0.0001), patient height (p=0.039), disease- free time (p=0.02) (**Table 3**). Only a trend was shown in difference in PTV value (p=0.05).

Linear regression analysis showed an inverse dependency of pCR on primary tumor volume and observed lymphopenia (p<0.05). Primary tumor downstaging had the largest impact on pCR (B=.37) with the level of statistical significance standing at less than p<0.001. Univariate analysis of variance showed the absence of heteroscedasticity in White’s test. It also showed a statistically significant effect on pCR achievement by lymphopenia at the conclusion of chemoradiation course (p=0.045), GTV (p=0.002), height (p=0.001), weight (p=0.023). pCR dependence on GTV and patient body weight were negative in terms of tumor response to delivered therapy.

**Figure 1:**
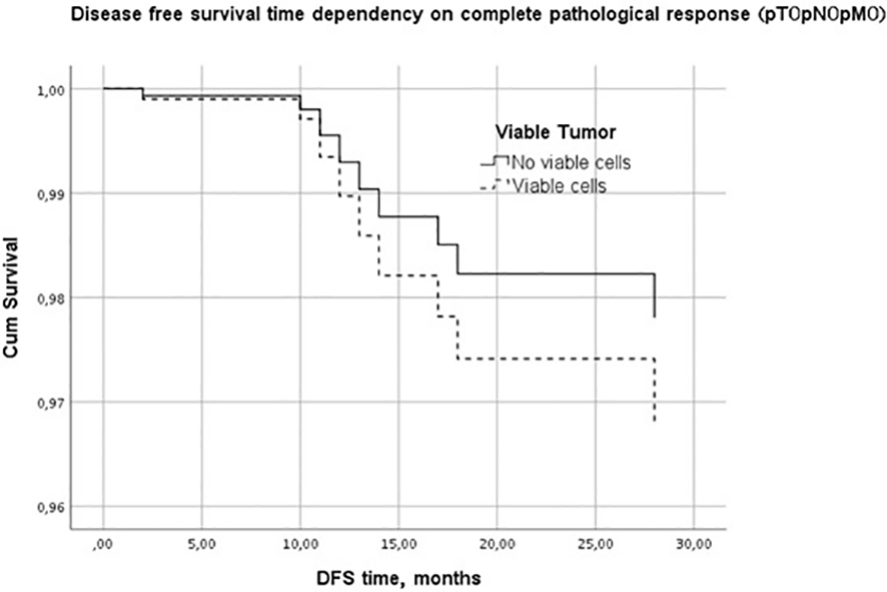
Disease free survival time dependency on pathological complete response.

Kaplan-Meier disease-free survival graph estimation test results showed no significant differences between the lymphopenic and non-lymphopenic groups, nor in the pCR or non-pCR groups, although visually the two survival lines were well separated (**Figure 1**). A multivariate Cox regression analysis was performed to further evaluate the effect of factors on the DFS period. It showed PTV as the most influential, DFS-modifying factor (p<0.01).

The factor analysis was performed to study the potential collinearity of variables affecting pCR and to clarify the model. It included patient age, smoking status, height, weight, as well as GTV, distance from the anal verge to the lower tumor margin in the rectal wall, lymphopenia, and tumor response to the neoadjuvant treatment. The analysis showed very low collinearity between the variables. However, statistical significance was observed in the correlation of lymphopenia and the following factors: height (p=0.02), distance to lower tumor margin (p=0.046), PTV45 (p=0.025), and achieved pCR (p=0.033). An inverted correlation was found between lymphopenia and the distance from anal verge to tumor margin and pCR. GTV was directly correlated with PTV (p<0.001) and inversely correlated with pCR (p=0.033). The PTV value directly correlated with body weight (p<0.001) and inversely correlated with the distance from the anal verge to the tumor (p<0.001). Patient body weight directly correlated with patient height (p<0.001).

## Discussion

Decreased lymphocyte levels in the bloodstream following radiation therapy were first described in the 1970s (23, 24), but the clinical significance of these declines has not been adequately investigated in rectal cancer. Therefore, the plan of our study was to focus on changes in lymphocytes’ levels in the bloodstream during chemoradiation, and their effect on preoperative treatment outcomes.

Microbiological studies have shown that sensitivity to radiation therapy depends not only on the biological characteristics of the tumor but also on its microenvironment (25, 26). Tumor reduction is affected by the immune response of the host in addition to the direct damage to the cancer cells caused by radiation (27). Additional studies have shown that the presence of immune cells in and around the tumor bed is associated with a better therapeutic response in colorectal cancer (28) and may be used as a tool to predict recurrence and survival in this cancer type. Nevertheless, there are studies that suggest a link between the level of lymphocytes in the bloodstream, which assume that blood cell count reflects host conditions and the effectiveness of radiation therapy for rectal cancer (16, 29). It should be noted that the association between lymphopenia after radiation therapy and recurrence rates has been examined in other cancers such as bladder (30) and head and neck tumors (31), but as aforementioned, not investigated adequately yet in rectal cancer.

A study published in the journal BMC Cancer in 2011 (16) examined the relationship between the effectiveness of radiation therapy and the levels of all blood cells withdrawn before and after treatment. Its results support the present study’s findings regarding lymphocyte levels. On the other hand, an investigation published in 2017 (32) offered contradictory findings, which suggested a decrease in the level of lymphocytes during preoperative treatment is associated with better tumor regression.

The present investigation found that the level of lymphocytes in the bloodstream decreases during radiation therapy and that this is an independent predictor of treatment efficacy and achievement of a pCR in LARC. Both absolute lymphopenia and relative lymphopenia were found to be associated with lower tumor regression rates. These findings emphasize the importance of follow-up throughout radiation therapy, while addressing the trend of lymphocytes levels during treatment and not just their absolute level at different time points.

Keeping in mind the potential influence of multicollinearity on study’s results, we evaluated each factor in terms of its pathophysiological impact on the processes within tumor, lymph nodes and volume of surrounding tissues. Using VMAT techniques for our patients, we produced the maximal gradient between PTV and surrounding pelvic bones to decrease adverse effects on bone marrow. We suggested that our strict bone marrow irradiation reduction policy is an appropriate way to reduce possible collinearity for study’s results.

The present research is a unique investigation that analyses variables throughout the course of antineoplastic treatment while normalizing individual values for each colorectal cancer patient. As there are few studies in the literature that have been conducted using the same methodology, it is critical to examine the lymphocyte level parameter in other cancers and larger sample numbers to establish whether the findings detailed here are random or not.

The information reported in this study may help medical oncologists predict the therapeutic response in this type of cancer. By monitoring lymphocyte levels during treatment and identifying those patients who may respond less well to, for example, the full preoperative treatment approach, they may be guided toward treatment therapeutic strategy adjustments and alternatives that will optimize outcomes. The current analysis was unable to find variables that predict the development of lymphopenia in different patients. Possible reasons for this may be insufficient sample size, analysis of non-real-time results (as the study is retrospective and relies on existing information), and the absence of a control group. More extensive prospective studies with larger sample sizes are needed before a more conclusive answer can be asserted to the question posed by this study – whether lymphopenia affects the response of radiation therapy to rectal cancer and what are the factors that can predict the development of lymphopenia in patients.

## Conclusion

Decreased levels of lymphocytes during preoperative CRT treatment of LARC are predictive of a non-pCR. It is associated with lower regression rates and may be a prognostic measure of therapeutic response. This study showed that weekly monitoring of the lymphocyte levels during preoperative treatment reflects the hematopoietic toxicity of radiation therapy and may also predict responsiveness to treatment. Monitoring the immune response to preoperative treatment by blood tests is a convenient and accessible clinical tool for identifying patients who may benefit from preoperative radiation therapy. It is also a practical way to diagnose patients with a lower likelihood of achieving a full response to treatment, thus creating opportunities to customize therapeutic approaches and offer adaptations and alternatives, such as full preoperative radiation therapy. Further prospective studies are needed to better understand the factors that could predict the development of lymphopenia in patients and thereby establish the means for treatment optimization.

## Data availability statement

The raw data supporting the conclusions of this article will be made available by the authors, without undue reservation.

## Ethics statement

The studies involving human participants were reviewed and approved by Rambam Healthcare Campus. Written informed consent for participation was not required for this study in accordance with the national legislation and the institutional requirements.

## Author contributions

ML, TT and SB contributed to conception and design of the study. ML organized the database and performed statistical analysis. TT wrote the manuscript. TT has Equal contribution and first authorship for this paper. ML - the concept and methodology formalization, data base creation, statistical analysis. All authors contributed to manuscript revision, read, and approved the submitted version.
